# Host phylogeny shapes viral transmission networks in an island ecosystem

**DOI:** 10.1038/s41559-023-02192-9

**Published:** 2023-09-07

**Authors:** Rebecca K. French, Sandra H. Anderson, Kristal E. Cain, Terry C. Greene, Maria Minor, Colin M. Miskelly, Jose M. Montoya, Michelle Wille, Chris G. Muller, Michael W. Taylor, Andrew Digby, Kākāpō Recovery Team, Jodie Crane, Galen Davitt, Daryl Eason, Petrus Hedman, Bronnie Jeynes, Scott Latimer, Sarah Little, Michael Mitchell, Jake Osborne, Brodie Philp, Alyssa Salton, Lydia Uddstrom, Deidre Vercoe, Alex Webster, Edward C. Holmes

**Affiliations:** 1 Sydney Institute for Infectious Diseases, School of Medical Sciences, The University of Sydney, Sydney, New South Wales Australia; 2 School of Biological Sciences, University of Auckland, Auckland, New Zealand; 3 Biodiversity Group, Department of Conservation, Christchurch, New Zealand; 4 School of Natural Sciences, Massey University, Palmerston North, New Zealand; 5 Te Papa Tongarewa Museum of New Zealand, Wellington, New Zealand; 6 Theoretical and Experimental Ecology Station, National Centre for Scientific Research (CNRS), Moulis, France; 7 Wildbase, School of Veterinary Science, Massey University, Palmerston North, New Zealand; 8 Kākāpō Recovery Team, Department of Conservation, Invercargill, New Zealand

**Keywords:** Viral evolution, Evolutionary genetics

## Abstract

Virus transmission between host species underpins disease emergence. Both host phylogenetic relatedness and aspects of their ecology, such as species interactions and predator–prey relationships, may govern rates and patterns of cross-species virus transmission and hence zoonotic risk. To address the impact of host phylogeny and ecology on virus diversity and evolution, we characterized the virome structure of a relatively isolated island ecological community in Fiordland, New Zealand, that are linked through a food web. We show that phylogenetic barriers that inhibited cross-species virus transmission occurred at the level of host phyla (between the Chordata, Arthropoda and Streptophyta) as well as at lower taxonomic levels. By contrast, host ecology, manifest as predator–prey interactions and diet, had a smaller influence on virome composition, especially at higher taxonomic levels. The virus–host community comprised a ‘small world’ network, in which hosts with a high diversity of viruses were more likely to acquire new viruses, and generalist viruses that infect multiple hosts were more likely to infect additional species compared to host specialist viruses. Such a highly connected ecological community increases the likelihood of cross-species virus transmission, particularly among closely related species, and suggests that host generalist viruses present the greatest risk of disease emergence.

## Abstract (web summary)

Sampling the viromes of vertebrates, arthropods and plants on an island ecosystem shows that viral transmission between species is strongly affected by phylogeny but less affected by predator–prey relationships and that generalist viruses pose the greatest zoonotic risk.

## Main

Cross-species virus transmission is a near-universal feature of viruses^1^. Determining how viruses move through ecosystems is central to understanding their occasional emergence as pathogens. Recent metagenomic studies suggest that only a small proportion of viruses cause serious disease and mortality, with apparently healthy wildlife species commonly infected by multiple viruses^2^. In reality, viruses are a key component of global ecosystems, regularly moving between species in the absence of overt disease^3^. As such, a full understanding of infectious disease emergence requires an ecosystem-level approach^3,4^, in which the viromes of entire interacting communities are investigated.

Two factors have been proposed to govern virus movement from one host species to another, shaping similarities and differences in virus composition among taxa and hence determining virome structure at the ecosystem scale. First, it is possible that host phylogenetic relatedness directly impacts the frequency and pattern of cross-species virus transmission^5^. Rates of cross-species virus transmission are expected to be higher among closely related host species, reflecting a greater similarity in the virus and host proteins that underpin successful virus–cell relationships such as host-cell binding^6^. A fundamental difference in virus–host cell relationships in part explains why viruses from vertebrates and invertebrates are usually phylogenetically distinct^7^, even though the latter are often dietary components of the former. A second proposed factor is that ecological properties of the host play a major role in virus movement between species by determining the probability of virus exposure^8^. Each time species interact, they provide opportunities for cross-species virus transmission. Hence, the more interactions, the greater the probability of host jumping. For example, changes in land use have increased human–animal interactions, driving disease emergence events in humans^9^. Predator–prey interactions are common exposure events, with the consumption of prey providing direct contact with viruses during digestion. Consequently, the structure of an ecosystem food web may have a large impact on the flow of viruses through communities. With the exception of marine microbial food webs in which viral lysis of microbial hosts impacts food web structure^10^, the prediction is yet to be tested.

**Extended Data Fig. 1:**
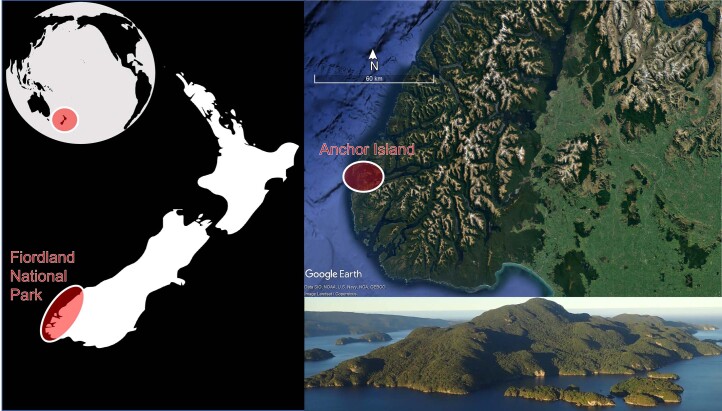
Map showing the location of Anchor Island, New Zealand. Left panel. Map of New Zealand showing the location of the Fiordland National Park. Adapted from SVG > countries navigation earth international (https://svgsilh.com/image/1504059.html) and SVG > south map New Zealand (https://svgsilh.com/image/309892.html?fbclid=IwAR1-73KzelpNXlyG92T6BaUtX5SHSsFcfEWynGhXTswBw1uE79UHYQFSgFI). Top right panel. Google Earth picture of the Fiordland National Park, New Zealand showing the position of Anchor Island. Google Earth citation provided in the picture. Photograph of Anchor Island taken by Dr. Rebecca French.

Metagenomic sequencing allows the entire virome of samples to be characterized^2^, allowing a more precise description of viral diversity and the increasingly rapid discovery of novel viruses^11^. Far less attention has been directed toward understanding how viruses move within ecosystems. Research on food webs including viruses has commonly focused on single pathogens of a species of interest^12^ or on the microbial subset of the food web^10^. The virome of an entire food web is yet to be characterized, in part because the large number of species in most ecological communities makes this impractical. However, in small island forest ecosystems, such as Pukenui/Anchor Island in the Fiordland region of southwestern New Zealand (Extended Data Fig. 1), the entire forest community is small enough that most broad taxonomic groups can be sampled, providing a snapshot of a food web virome.

The unique evolution of New Zealand wildlife (that is, the almost complete absence of native terrestrial mammals) means that unlike other forest ecosystems the Pukenui/Anchor Island ecological community is dominated by birds, with no terrestrial mammals, and only one species of reptile. However, despite being small, isolated and having a low diversity (relative to forest ecosystems outside New Zealand), this community contains a breadth of ecological niches, including a variety of diets: carnivores, insectivores, plant-eaters (including herbivores, frugivores, nectarivores and granivores), piscivores and omnivores. This ecological community also contains threatened species that may be vulnerable to disease emergence via cross-species virus transmission: for example, the critically endangered kākāpō (*Strigops habroptila*), endangered mohua (*Mohoua ochrocephala*) and critically endangered Te Kakahu/Chalky Island skink (*Oligosoma tekakahu*). The Pukenui/Anchor Island community also has a relative decoupling of phylogenetic relatedness and ecological niches. Some species that are distantly related (that is, from different phylogenetic orders) possess a similar ecological niche and regularly interact. For example, yellow-crowned parakeets (*Cyanoramphus auriceps*, kākāriki) were frequently observed with passerines (brown creeper *Mohoua novaeseelandiae*, mohua, and the grey warbler *Gerygone igata*, riroriro), travelling and foraging in multi-species flocks (R.K.F. personal observation), as observed elsewhere in New Zealand^13^.

**Fig. 1:**
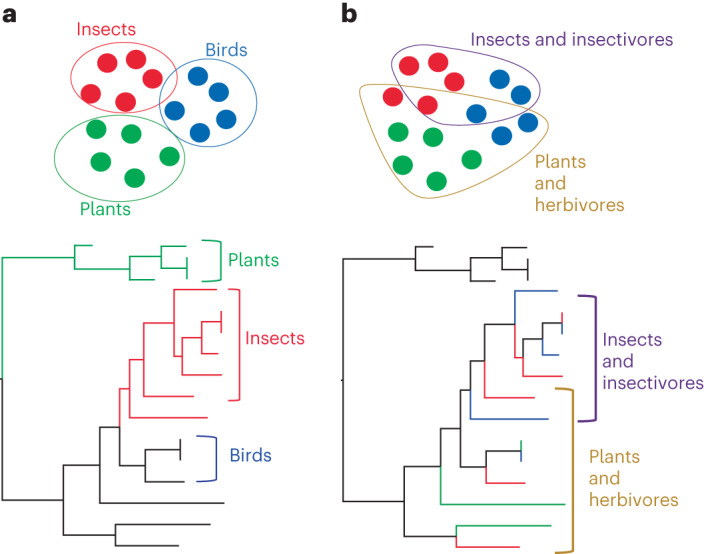
Expected impact of two main drivers of virus diversity on the structure of virus phylogenies. **a**, Host phylogenetic relationships drive virus diversity. **b**, Host feeding ecology drives virus diversity. Circles denote hypothetical clusters of hosts with similar viromes: that is, dots (hosts) within a circle have more viruses in common than to other dots outside the circle.

We used metatranscriptomic (that is, total RNA) sequencing to document the virome of each host in the community, including viruses that directly infect the host, bacteriophage and those present in the host diet. Hence, we use the total viral diversity of each host, rather than only those viruses that have established a true infection, to provide a broader view of virus transmission. Viruses that have established infection in their hosts could represent transient spill-over events or sustained cross-species transmissions and result in acute or chronic infections. If host phylogeny were the key driver of viral diversity, we would expect viromes to cluster according to the major host phyla (for example, Chordata, Arthropoda and Streptophyta), as well as at lower taxonomic levels, with more closely related hosts having more similar viromes than hosts that are more distantly related (Fig. 1a). By contrast, if host ecology were the main driver of virus diversity, we would expect viromes to cluster according to major dietary associations, with hosts that have similar diets possessing similar viromes, and predators and prey clustering together (Fig. 1b).

Our sampling of the Pukenui/Anchor Island forest community included all key vertebrate species in addition to representative invertebrates and plants (Supplementary Table 1). The sampled community comprised five host phyla, 13 classes and 37 orders and was sampled over a 4 week period. Thus, this study is necessarily a ‘snapshot’ of the viral community at a single time point. We conducted diversity and network analyses to determine the importance of host phylogeny and host ecology on viral diversity at the virus family level.

## Results

### Viruses predominantly cluster according to host phylogeny

**Fig. 2:**
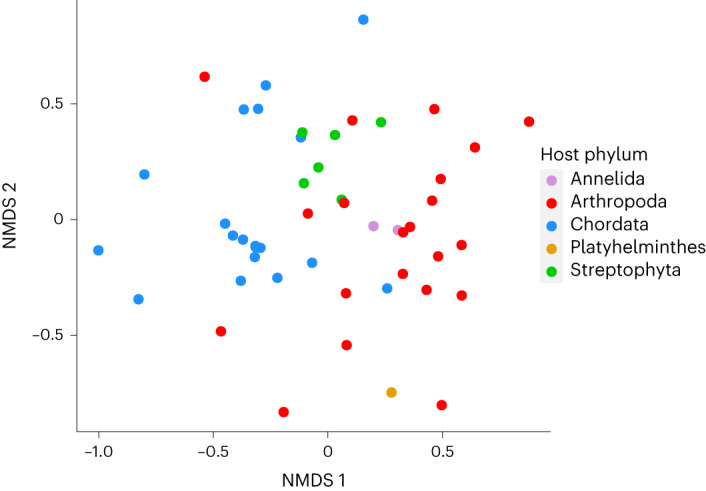
The similarity and dissimilarity of viral communities among host taxa. The first two dimensions of a three-dimensional non-metric multidimensional scaling (NMDS) plot show that virus communities at the family level cluster according to host phyla. Axes refer to the dimensions of the NMDS; stress = 0.196, *n* = 49.

We identified 16,633 viral sequences (assembled contigs) with an abundance of 360 million reads from a total of 112 different viral families. The number of viral families in each library ranged from 3 to 45, with a mean of 18.9 (±11.0 s.d.). Viral richness was dependent on host taxonomy (phylum *P* = 0.01, class *P* = 0.03 and order *P* = 1.05 × 10^−14^, *n* = 49). Non-metric multidimensional scaling plots similarly revealed that, overall, viral communities clustered to the level of host phyla (Fig. 2), which was confirmed using pairwise permutational multivariate analysis of variance (PERMANOVA) tests (Supplementary Table 2). All comparisons between Chordata, Arthropoda and Streptophyta were significant (*P* < 0.05, *n* = 49). In addition, host phylogenetic order explained the most variation between viral communities (*R*^2^ = 0.80) compared to host phylum (*R*^2^ = 0.13) and class (*R*^2^ = 0.30), when used as the dependent variable in the PERMANOVA test (*n* = 49).

Within Chordata (for which most host resolution is available), we used host order and host diet to compare how much variation in the viromes is explained by each factor. Host diet was described using two separate binary factors based on the primary diet types (Supplementary Table 1)—‘insectivore’ (yes or no) and ‘plant-eater’ (yes or no). Nectar, fruit and seed eaters were assigned as plant-eaters. Omnivores that feed on both invertebrates and plants were assigned as both an insectivore and a plant-eater. Carnivores and piscivores were assigned as neither an insectivore nor a plant-eater. In this case, viral richness was dependent on host order (*P* < 2 × 10^−16^, *n* = 19) but not whether the hosts ate insects (*P* = 0.9, *n* = 19) or plants (*P* = 0.3, *n* = 19). When controlling for host order, PERMANOVA tests revealed that insectivores had significantly different viromes from non-insectivores, and plant-eaters had significantly different viromes from non-plant-eaters (*P* < 0.05, *n* = 19, Supplementary Table 2). However, host order explained much more of the variation (*R*^2^ = 0.47) than diet (insectivore *R*^2^ = 0.06, plant-eater *R*^2^ = 0.07). A similar result was obtained when including only viruses likely to infect chordates, with all comparisons significant (*P* < 0.05, *n* = 19), and host order explaining more variation (*R*^2^ = 0.48) than host diet (insectivore *R*^2^ = 0.06, plant-eater *R*^2^ = 0.06).

To further determine the relative impact of host phylogeny and ecology on virus community composition within the Chordata, we assessed the degree of correlation between three dissimilarity matrices: viral community (a Bray–Curtis dissimilarity matrix), host relatedness (generated using the sum of branch lengths from a host phylogeny) and host ecological similarity (using the dietary factors described above). In this case, host ecological similarity and viral community were significantly correlated (Mantel statistic *R* = 0.25, *P* = 0.009), even after controlling for host relatedness (Mantel statistic *R* = 0.25, *P* = 0.01). By contrast, there was no significant correlation between host relatedness and viral community (Mantel statistic *R* = −0.025, *P* = 0.64).

**Fig. 3:**
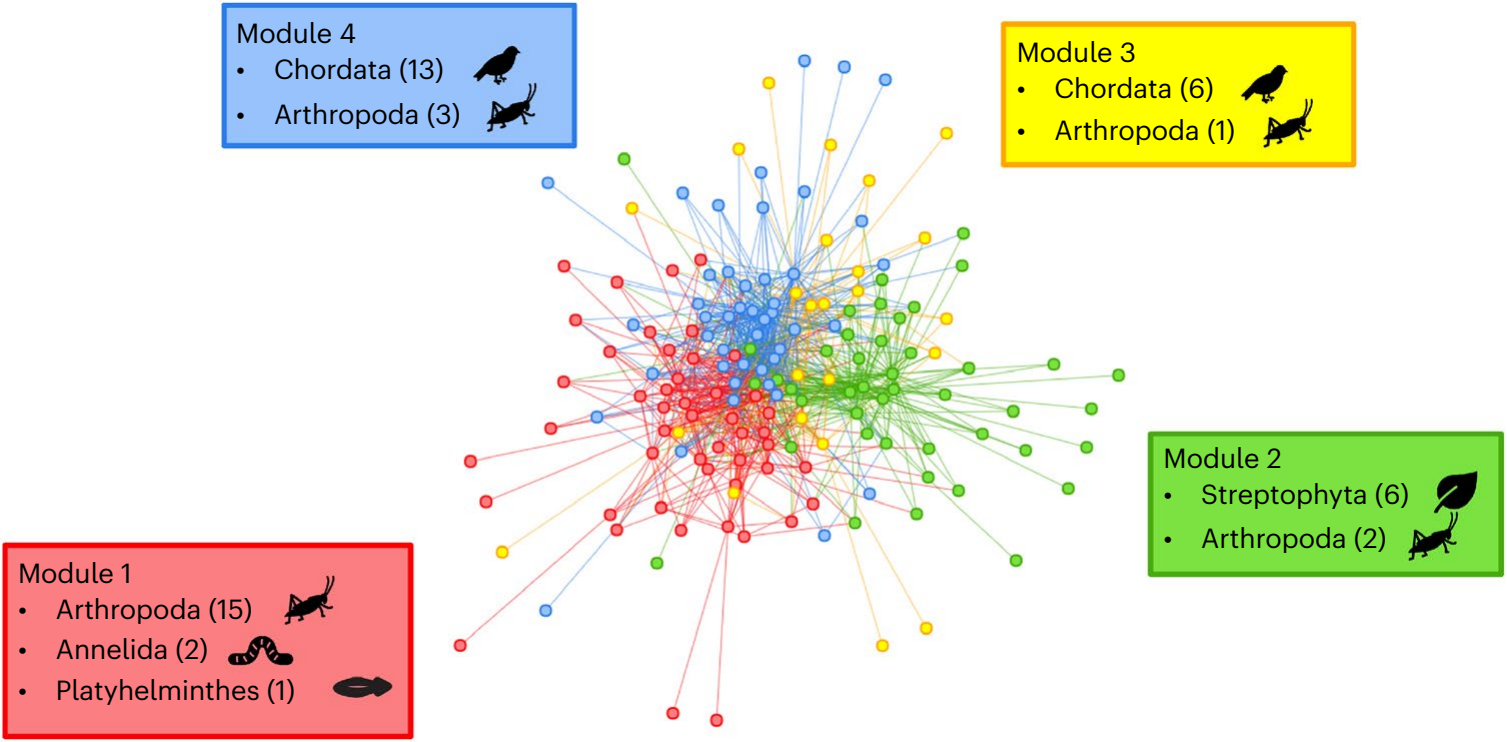
Host–virome network (bipartite) displayed using the Fruchterman–Reingold layout. The modules are shown by node colour. Nodes include both host library and virus families. The boxes show the host makeup of each module, with the number of hosts belonging to each host phylum shown in parenthesis.

To explore the broad clustering of viral communities by host phylogeny, we created a bipartite network (herein referred to as the ‘host–virome network’) and identified four modules using a community detection algorithm. Notably, the modules follow host phylogenetic groupings, with a Pearson’s chi-squared test of independence indicating that host phylum and module were highly correlated (*χ*^2^ = 68.4, d.f. = 12, *P* = 6.4 × 10^8^). Module 1 contained only invertebrates (arthropods, annelids and a platyhelminth), module 2 predominately comprised plants (75% plants and 25% invertebrates), while modules 3 and 4 largely comprised chordates (86% and 81% chordates, 14% and 19% invertebrates, respectively) (Fig. 3). All modules contained both DNA and RNA viruses. Module 3 comprised mostly bacteriophage, while the other modules were dominated by viruses that infect the hosts in those modules.

Modules also had differing levels of host and virus richness, with module 1 containing the highest number of hosts (18), module 2 containing the highest number of virus families (36) and module 3 containing the lowest number of both hosts (7) and virus families (17). The communities within each module were significantly different from one another (*P* < 0.05, *n* = 49, pairwise PERMANOVA test; Supplementary Table 2), which was robust to rarefication (Supplementary Table 3). Interestingly, the two modules containing chordates (modules 3 and 4) had the smallest difference in communities (Supplementary Table 2), although still statistically significant.

### Network structure

**Fig. 4:**
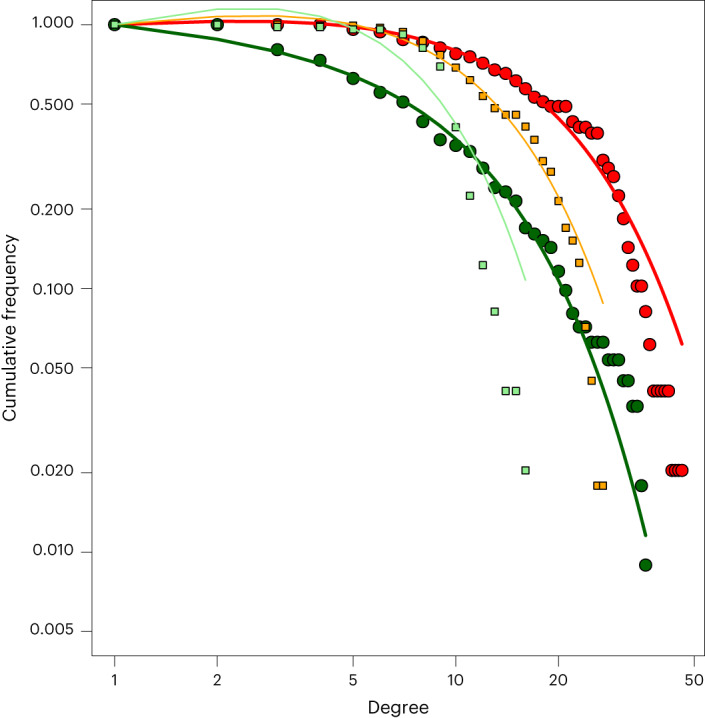
The degree distribution of the host–virome network (large circles) and the null network (small squares), shown as the cumulative probability of finding a virus family in the network with *k* or less-associated hosts (*Pc*(*k*)). The colour denotes node type, with red and orange referring to host nodes and the dark and light green the virus family nodes, in the host–virome network and null network, respectively.

We next analysed the structure of the host–virome network using the degree distribution (that is, the distribution of the number of links between nodes) in comparison to a network generated with a null model (a bipartite network with the same number of nodes and links, randomly assigned). The cumulative degree distributions for the host and virus nodes followed a truncated power-law distribution (Kolmogorov–Smirnov test *P* > 0.05) of *Pc*(*k*) = *k*^0.08^*k*^−(*k*/57)^ for the hosts and *Pc*(*k*) = *k*^−0.12^*k*^−(*k*/32)^ for the virus families, as shown by the fit lines in Fig. 4. *Pc*, the cumulative probability; *k*, the number of viruses/host nodes. The null networks also follow truncated power-law distributions of *Pc*(*k*) = *k*^0.17^ *k*^−(*k*/30)^ and *Pc*(*k*) = *k*^0.34^*k*^−(*k*/14)^ but with lower cut-off values than the host–virome network. Viruses with few connections had fewer than the random expectation, while viruses with more connections had more than expected by chance, shown by the null and virus distributions intersecting at approximately 10 links (Fig. 4). By contrast, all hosts had systematically more connections than the random expectation.

Across node level properties, degrees, betweenness and eigenvector centrality were significantly dependent on host taxonomy across all levels (phylum degree *P* = 0.0013, betweenness *P* = 0.0026, eigenvector centrality *P* = 0.041; class degree *P* = 0.003, betweenness *P* = 3.8 × 10^−16^, eigenvector centrality *P* = 0.039; order degree *P* < 2 × 10^−16^, betweenness *P* = 0.0012, eigenvector centrality *P* < 2 × 10^−16^; *n* = 49). Within the Chordata, host order but not host diet significantly impacted degree, betweenness and eigenvector centrality (Supplementary Table 4).

### Potential for virus movement within the network

**Fig. 5:**
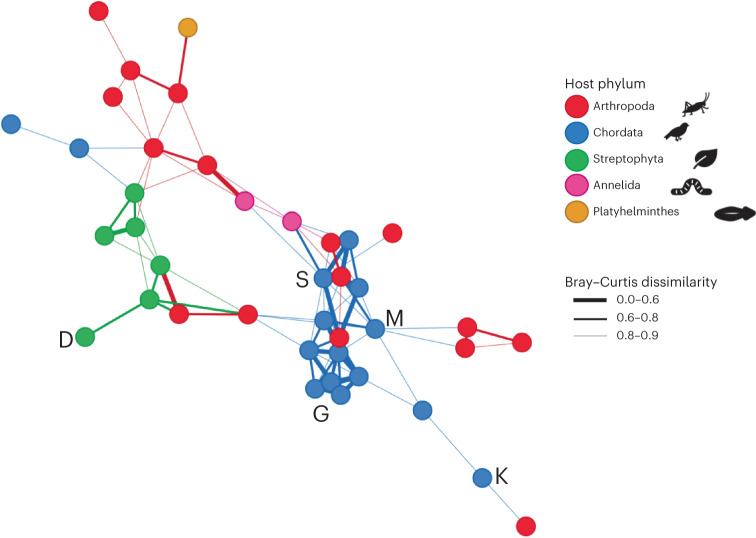
Host community network (unipartite) displayed using the Fruchterman–Reingold layout. Nodes are connected to other nodes if they have a dissimilarity value of less than 0.9. The thickness of the line shows the level of dissimilarity. The Bray–Curtis dissimilarity statistic ranges from 0 to 1, with 0 meaning two hosts have identical viromes at the viral family level, and 1 meaning two hosts have no viral families in common. The letters refer to species of interest: D, moss; G, grey warbler; K, kākāpō; M, mohua; S, Te Kakahu skink.

To examine the strength of connections between hosts that shared viruses, we created a unipartite network (that is, links between hosts with shared virus families, where a link is a connection between two hosts) based on the Bray–Curtis dissimilarity matrix, which we refer to here as the ‘host community network’ (Fig. 5). This network had a high level of connectivity between hosts and within host phyla, with the connections across host phyla generally weaker (that is, a higher Bray–Curtis value). The maximum shortest path was eight, such that the most distantly connected nodes were still only eight links (connections between hosts) away from one another. The mean shortest path distance was only 3.19, such that on average each node is 3–4 links away from every other node. Also noteworthy was a key cluster containing predominately chordates with a high number of strong connections. The two host species with very high richness (moss (*Dicranoloma billardierei*) and grey warbler, with over 40 viral families in each) differed in their connectivity in the network (Fig. 5), with the moss having a low number of connections to other nodes (1), while the grey warbler had a high number of connections (6) and was part of the chordate cluster.

In comparison to 1,000 randomly generated null networks, the transitivity ratio (that is, the probability that the adjacent nodes are connected, expressed as a ratio comparing the null and host community network values) was high (3.1), while the path length ratio (the smallest number of links between each node) was similar (1.1), with a ‘small-worldness’ index of 2.7. This signifies a small world network: both highly clustered and highly connected. Most nodes are a small number of links away from every other node, enabling viruses to easily move between species^14^. Our analysis also revealed that if a virus were to move through this network, it would first move within a host phylum, then most likely from plants to arthropods, and to/from arthropods and chordates. Strikingly, direct links between plants and chordates were far less common, which may reflect the relatively small number of chordates on the island that eat plants, compared to those that eat invertebrates (Supplementary Table 1).

### Host ecology affects viral diversity at smaller scales

**Fig. 6:**
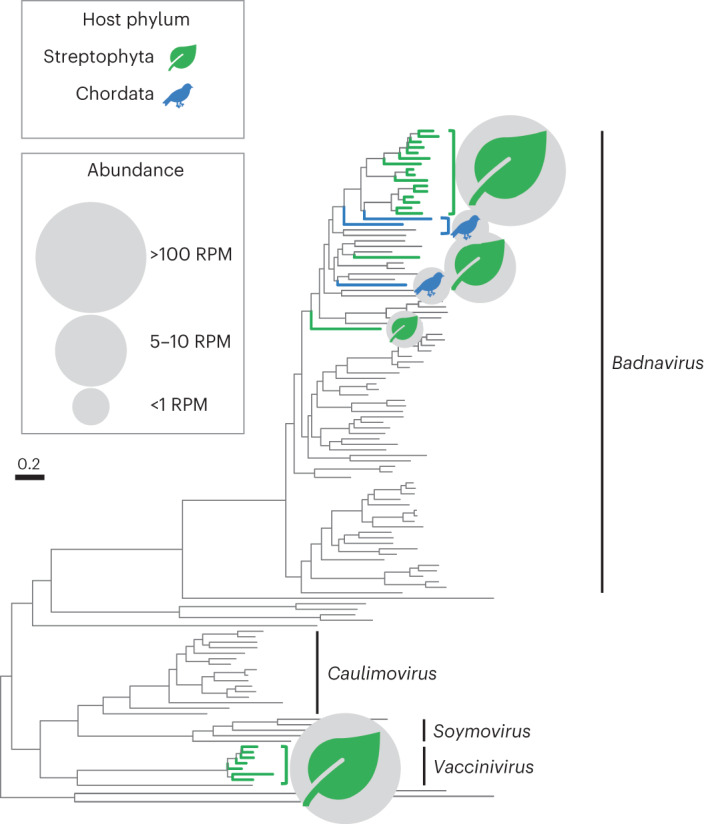
Virus diversity at the species level. Phylogeny of the *Caulimoviridae* (module 2). The colours and symbols correspond to host phyla: green leaf, Streptophyta; blue bird, Chordata. All unmarked viruses have plant hosts. The abundance of viruses in each phylum is shown in the key inset, expressed as RPM. Branches are scaled according to the number of amino acid substitutions per site, shown in the scale bar. The tree is midpoint rooted for display purposes only. Detailed individual phylogenies, sequence alignments and information including the genes used, alignment length, percentage identity and number of sequences can be found in Extended Data Figs. 2–5 and Supplementary Tables 6–7.

**Extended Data Fig. 2:**
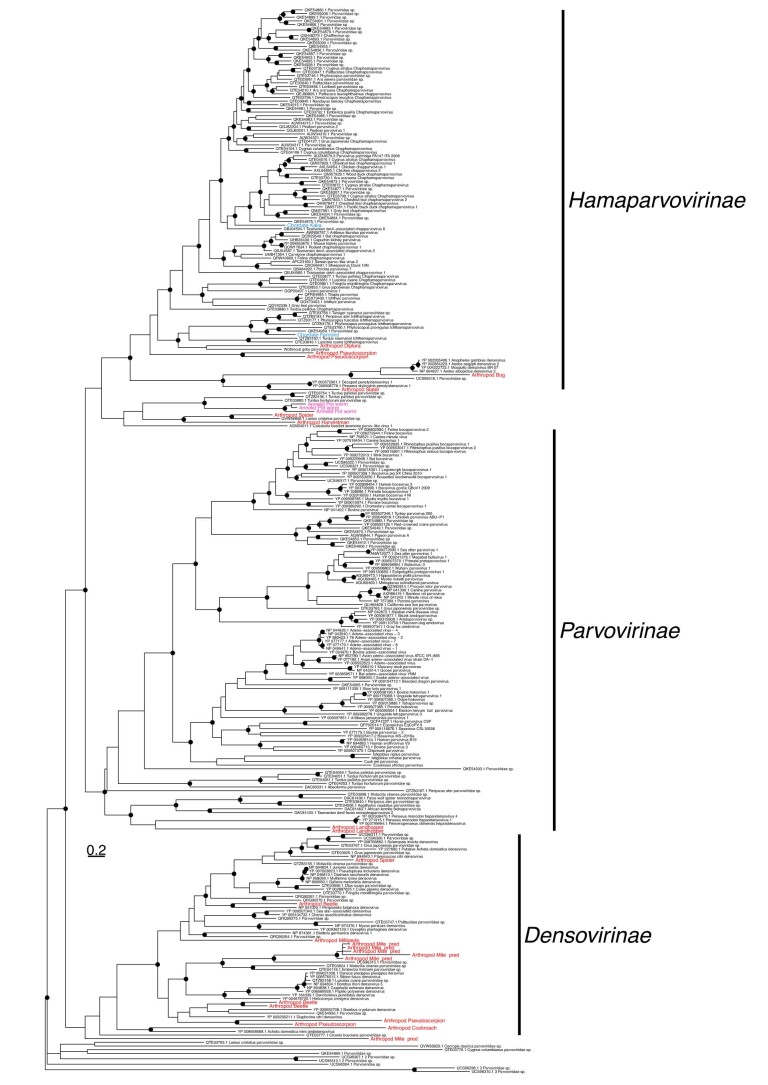
Phylogenetic tree of the *Parvoviridae* based on the non-structural protein 1 gene (alignment length 584 amino acids) (related to Fig. 6). The colours correspond to host phyla, blue = Chordata, red = Arthropoda, pink = Annelida. Viruses from this study are labelled with their phylum and host common name (see Supplementary Table 1 for details on each host). Branches are scaled according to the number of amino acid substitutions per site, shown in the scale bar. Black circles on nodes show bootstrap support values of more than 90%. The tree is midpoint rooted for display purposes only.

**Extended Data Fig. 3:**
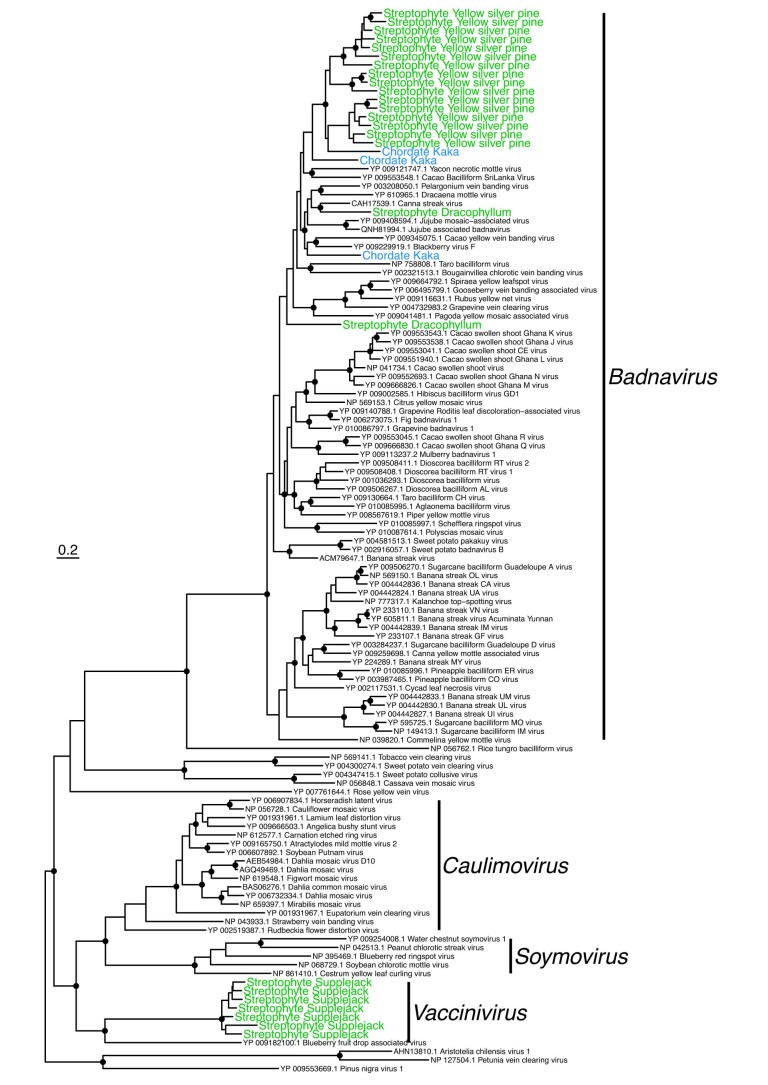
Phylogenetic tree of the *Caulimoviridae* based on the polyprotein gene (alignment length 938 amino acids) (related to Fig. 6). The colours correspond to host phyla, green = Streptophyta, blue = Chordata. Viruses from this study are labelled with their phylum and host common name (see Supplementary Table 1 for details on each host). Branches are scaled according to the number of amino acid substitutions per site, shown in the scale bar. Black circles on nodes show bootstrap support values of more than 90%. The tree is midpoint rooted for display purposes only.

**Extended Data Fig. 4:**
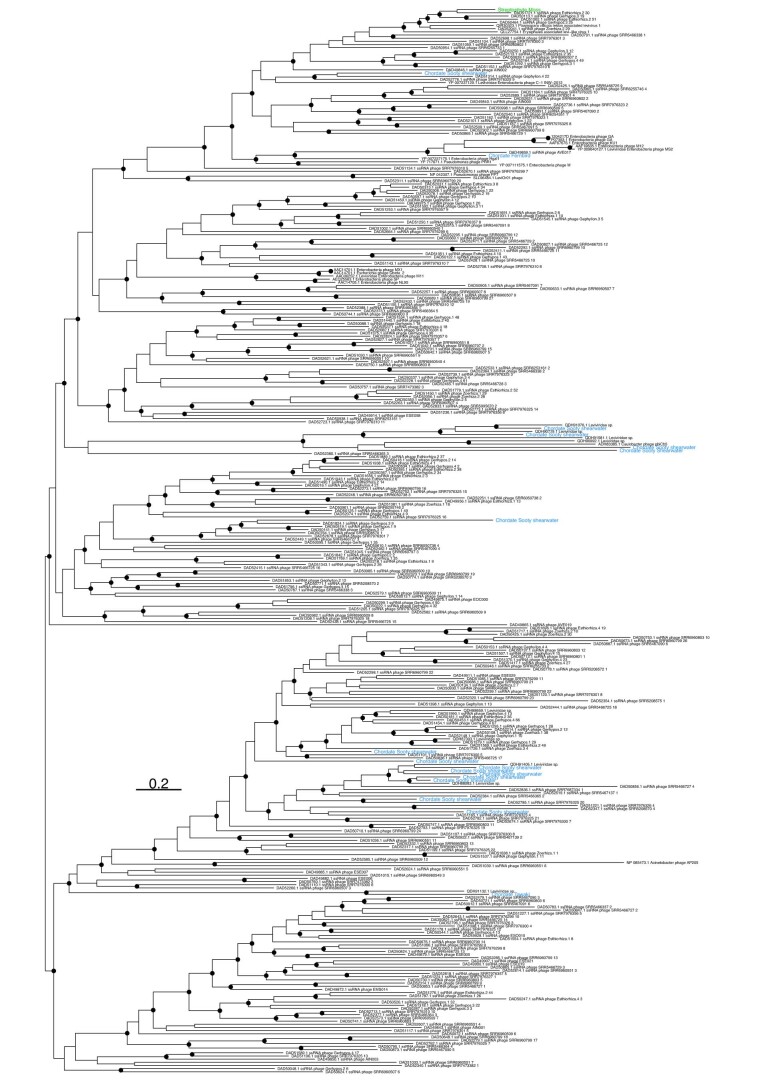
Phylogenetic tree of the *Fiersviridae* based on the RNA-dependent RNA polymerase gene (alignment length 453 amino acids) (related to Fig. 6). The colours correspond to host phyla, green = Streptophyta, blue = Chordata. Viruses from this study are labelled with their phylum and host common name (see Supplementary Table 1 for details on each host). Branches are scaled according to the number of amino acid substitutions per site, shown in the scale bar. Black circles on nodes show bootstrap support values of more than 90%. The tree is midpoint rooted for display purposes only.

**Extended Data Fig. 5:**
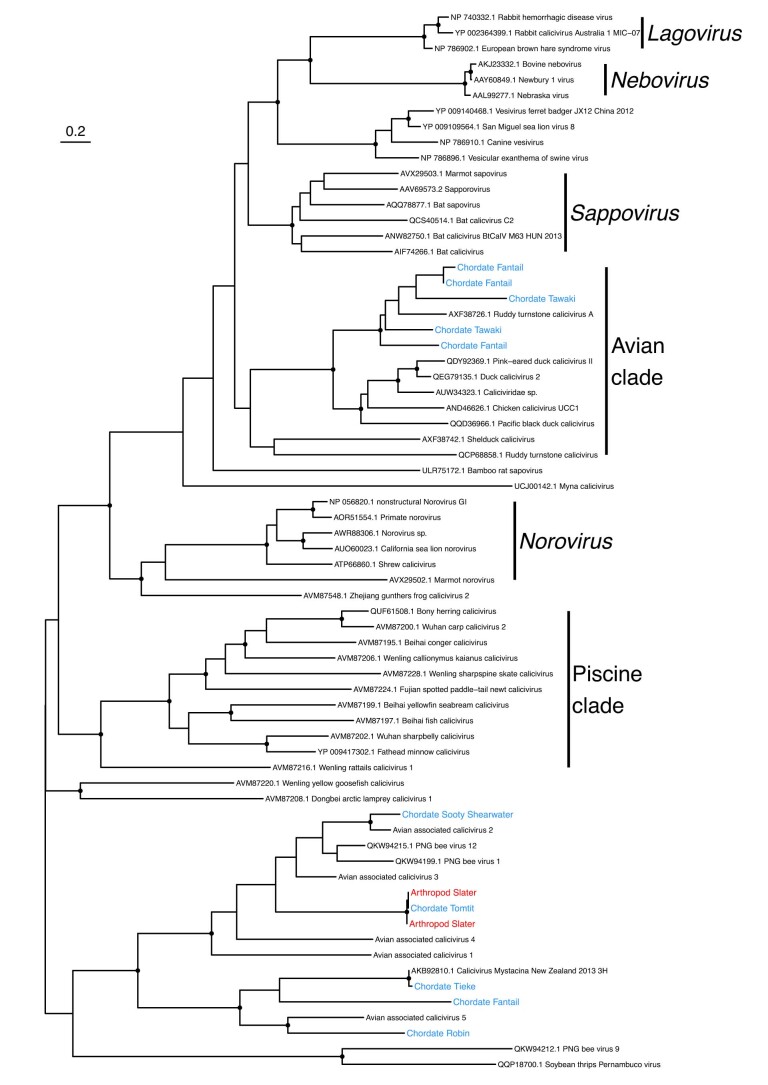
Phylogenetic tree of the *Caliciviridae* based on the polyprotein gene (alignment length 1313 amino acids) (related to Fig. 6). The colours correspond to host phyla, red = Arthropoda, blue = Chordata. Viruses from this study are labelled with their phylum and host common name (see Supplementary Table 1 for details on each host). Branches are scaled according to the number of amino acid substitutions per site, shown in the scale bar. Black circles on nodes show bootstrap support values of more than 90%. The tree is midpoint rooted for display purposes only.

To examine the diversity of virus species within and across host phyla, we conducted a phylogenetic analysis for one virus family per module: *Parvoviridae* (module 1), *Caulimoviridae* (module 2), *Fiersviridae* (module 3) and *Caliciviridae* (module 4), chosen for their high viral diversity across multiple host species (Fig. 6, Extended Data Figs. 2–5 and Supplementary Tables 5–7). In general, there was a high degree of virus clustering by host phyla, with each module containing a high richness and abundance of virus species from their predominant host. However, there was also evidence of host ecology (that is, predator–prey interactions) influencing virus diversity, with closely related/identical viruses found in distantly related hosts. For example, we identified three viruses in the kākā (parrot) library from the plant virus family *Caulimoviridae* that were closely related to members of the viral genus *Badnavirus* found in the plants sampled here (Fig. 6). This pattern is indicative of a food web interaction in which the parrot consumed the plants. Similarly, we found near-identical (>99% at the amino acid level) members of the *Caliciviridae* in a miromiro/tomtit (passerine, *Petroica macrocephala*) and slater/woodlouse (arthropod, Isopoda species) library, strongly indicating a food web interaction (Extended Data Fig. 4). As the arthropod is a detritivore and the bird predominately insectivorous, the interaction could have occurred in either or both directions.

## Discussion

We used a metatranscriptomic approach to reveal the drivers of virome structure based on a ‘snapshot’ of the viral community on a relatively isolated island. We achieved this by creating the (bipartite) host–virome network based on the presence or absence of each virus family in each host library, and the (unipartite) host community network based on the level of virome similarity between hosts.

This analysis revealed a strong effect of host phylogeny on the host–virome network, with more viral families shared within than between host phyla. Host taxonomy also impacted viral richness and node level properties within the host–virome network. To our knowledge, this is the first such evidence from an ecosystems-scale analysis, and hence supports analyses of specific viral families and hosts that have shown host phylogenetic distance to be a key constraint on the cross-species transmission of viruses and hence on disease emergence^5,15^. Similarly, host phylogeny plays an important role in shaping the diversity of bacteria and eukaryotic parasites^16,17^, such that it likely has wide-ranging impacts on microbial diversity. Despite this, the strength of the host phylogenetic trend observed here is surprising given that the viromes characterized comprised all the actively transcribed viruses identified, not just those directly infecting the host in question. There were, necessarily, differences in sampling method between chordates (cloacal swab), invertebrates (body tissue) and plants (leaf tissue) which, while unavoidable, could have resulted in artificial similarities in the virome between similar sample types. However, cloacal swabs should have biased the results toward food web interactions as they represent a sample of the host digestive tract which often contains diet-associated viruses^18^. Moreover, we found significant differences between viral communities at lower host phylogenetic levels (class and order) where the sampling method was consistent, suggesting the differences observed were not due to the sampling method.

That viruses generally cluster by host phylogeny means that virus–host co-divergence has a major impact in shaping virome structures at deep evolutionary scales. Phylogenetic barriers likely prevent frequent cross-species virus transmission between phyla because this process can only occur between species with similar virus–cell interactions, such as receptor binding in the case of animal viruses. This results in a mixture of host–virus co-divergence at deeper taxonomic levels and cross-species transmission at shallower taxonomic boundaries, with the combination of both processes meaning that more closely related species have generally similar viromes. For example, although host phylogeny at the order level had a stronger impact than host ecology on the virome community within the Chordata, only virus composition and host diet had significantly correlated dissimilarity matrices. Indeed, phylogenetic analysis at the level of virus species provided direct evidence for cross-species transmission with, for example, a cluster of closely related caliciviruses in both the fantail (*Rhipidura fuliginosa*, passerine) and the tawaki (*Eudyptes pachyrhynchus*, penguin) (Extended Data Fig. 4). This accords with comparative studies that have revealed relatively weak host–virus co-divergence among viruses sampled from different host classes^19,20^.

Within the Chordata, predator–prey interactions were not associated with high levels of cross-species viral transmission in this island community, again likely reflecting phylogenetic constraints. It is possible, however, that more predator–prey viral transmissions would be detected in ecological communities where such interactions occur more frequently between closely related species, thereby reducing the effect of host phylogeny, and that viruses absent from chordate cloacal swabs may be subject to different evolutionary patterns. Indeed, studies of the human gut have provided limited evidence for host diet impacting virome structure^21^. However, other aspects of host ecology such as location, age and behaviour (within a narrower host range than our study) all influence host viromes, although to a lesser extent than phylogeny^22,23^. Despite the limited influence of host ecology on viral diversity, we found clear examples of host predator–prey interactions at the level of virus species, showing that viruses do move between species via these processes. Overall, our results suggest that virus traffic from predator–prey interactions between distantly related species is only transient (that is, the virus is only present for a short time in the predator’s digestive system) and hence unlikely to result in productive infections. Our results therefore suggest that increases in host connectivity via predator–prey interactions would not result in increased rates of infection.

Both the host–virome network (bipartite) and host community network (unipartite) had similar structures to those observed in other ecological networks, including a truncated power-law distribution and small world network, structures that are commonly found in food-web and plant–pollinator networks^24,25^, suggesting they have similar constraints in network construction. Our host–virome network followed a truncated power-law distribution that had lower cut-off values than expected by chance. This suggests that the structure is influenced by assembly mechanisms (that is, ecological processes or phylogenetic traits of the host or virus that alter how the network is constructed). Power-law distributions occur when nodes are likely to get more links the greater the number they already have, such that the ‘rich get richer’^24^. Accordingly, hosts that already have a high diversity of viruses are more likely to acquire new viruses than hosts with a low diversity of viruses. This could be partly driven by species abundance^24^, with more abundant host species more likely to be exposed to viruses within the network. Similarly, viruses with many hosts are more likely to acquire more hosts, which may inform zoonotic risk assessments^26^. In particular, rather than assigning the highest risk score to viruses that are similar to those that have already emerged, it may be of greater utility to give preference to the most generalist viruses. This power-law distribution is truncated when the underlying model no longer accurately predicts the distribution beyond a certain cut-off, effectively preventing the rich from getting richer beyond that point. The higher this cut-off, the greater the number of highly connected hosts and viruses^27^. In our models, this cut-off value was higher than for the null network, indicating that the host–virome network contains more highly connected hosts and viruses (that is, generalists) than expected by chance. Truncated power-law distributions are often found in mutualistic networks (for example, plant–pollinators), with the truncation thought to be due to ‘forbidden’—physically impossible—links^28^. In our network, forbidden links could be due to phylogenetic barriers preventing cross-species transmission.

The host community network (unipartite) was a ‘small world’ network. Although clear clusters emerge, most nodes (hosts) can be reached via a small number of connections to other nodes^25^. This is common in ecological networks, with most species only two links apart on average in complex food webs^29^. The combination of strong modularity in our host virome network and high connectivity in our host community network implies that a pathogenic virus could rapidly move through the network, particularly once the virus crossed from one module to another, without requiring a direct consumer relationship between hosts. The host community network showed an especially strong clustering within chordates, with a high degree of similarity between hosts. This cluster included endangered species such as the mohua and Te Kakahu skink, suggesting that on Pukenui/Anchor Island these species are vulnerable to disease emergence from other chordates within that cluster. By contrast, the kākāpō was less closely connected to this cluster, suggesting it may be less vulnerable.

Our study shows that the phylogenetic relatedness of hosts is a strong driver of viral diversity in this ecological community. Phylogenetic barriers between distantly related hosts may prevent frequent virus movement despite exposure events via predator–prey interactions. The ecological community studied is highly connected, presenting risks for disease emergence in vulnerable species. This work sheds light on the processes that dictate viral movement through ecosystems and could be expanded to include sampling over multiple time points to further understand these processes. More research is needed on how the disruption of these networks impacts disease emergence.

## Methods

### Study location

Pukenui/Anchor Island is a small island (11.4 km^2^), located in Dusky Sound, Fiordland, New Zealand (Extended Data Fig. 1). The island is part of the largely uninhabited Fiordland National Park (over 12,000 km^2^) on the south-west coast of the South Island and is over 80 km to the nearest township by air. Following the eradication of invasive mammals in the early 2000s, the island became a key habitat for endangered native species, including the kākāpō (*S. habroptila*). The island is also an important habitat for seabirds that nest in the forest, including the tawaki/Fiordland crested penguin (*E. pachyrhynchus*). The temperate rainforest consists predominately of beech and podocarp (conifer) trees, with an understory/forest floor including shrubs, vines and mosses. To our knowledge, the only non-native permanent inhabitant of the island is the invasive German wasp (*Vespula germanica*).

### Sample collection

This research was conducted under a Department of Conservation Wildlife Act Authority authorization number 86173-FAU and authority for research and/or collection of material on public conservation land authorization number 86172-RES and had ethics approval from the University of Auckland reference number 002198.

Fieldwork was undertaken on Anchor Island, Fiordland, New Zealand, between 17 February and 14 March 2021. The 18 bird and 1 skink species were caught using four different methods, depending on the species in question. Small, flighted birds were caught using low-canopy mist netting, while larger flighted birds were caught with high-canopy mist nets. Bird calls were used to attract the birds to the area and into the nets. Non-flying birds were caught by hand or hand net. Skinks were caught using gee-minnow traps. Once caught, the animals were weighed, and a cloacal swab was taken, using a sterile nylon flocked swab FLOQswab (Copan), either mini-tip or regular tip depending on the size of the animal. The entire tip of the swab was inserted into the cloaca and swabbed with two to four circular motions while applying gentle pressure against the mucosal surfaces. The swab was then cut using scissors sterilized with 70% alcohol and placed into a tube with 1 ml of RNAlater. Samples were kept at −20 °C for the duration of the fieldwork, then frozen at −80 °C.

Leaves were collected from each plant species by cutting the stem with sterile scissors and placing the leaves into a sterile collection bag (one bag per individual plant). At the fieldwork base, a leaf from each individual plant was chopped into approximately 5 mm × 5 mm pieces and placed into 1 ml of RNAlater. The total volume of the solution and plant material was no more than 1.3 ml, to ensure preservation of all the RNA. To ensure the RNAlater permeated into the tissue, samples were left at 4 °C for approximately 12 h before being transferred to −20 °C. Samples were kept at −20 °C for the duration of the fieldwork, then frozen at −80 °C.

Invertebrates were collected by manual search and by extraction from soil. At five sites on Anchor Island, the area within a 5 m × 5 m square was intensively searched for invertebrates (on vegetation, under logs, under bark and so on). When an invertebrate was found, it was placed alive into a sterile pottle with damp moss or leaf litter from the site. At the same site, a sterilized spade was used to cut a soil core approximately 2 l in volume. The core was placed into a sterilized 2 l container. The invertebrates and soil cores were kept at 4 °C for the duration of the fieldwork. They were then transferred to Massey University, New Zealand. The invertebrates collected by hand were examined live under a dissecting microscope using sterile tools and identified to the lowest classification level possible (highest = order level, lowest = species level). Invertebrates from the soil cores were extracted using Berlese funnels into RNAlater and identified in the same way. Once identified, the invertebrates were individually stored at −80 °C.

### RNA extraction

#### Cloacal swabs

RNA was extracted using the RNeasy plus mini extraction kit (Qiagen) and QIAshredders (Qiagen). The tube containing the swab in RNAlater was thawed and the swab removed from the tube using sterile forceps and placed in 600 µl of extraction buffer. The swab and buffer were vortexed for 2 min at maximum speed. The swab and buffer were then placed into a QIAshredder and centrifuged for 5 min at maximum speed. The flowthrough was retained (avoiding the cell debris pellet) and used in the extraction following the standard protocol in the kit. The RNA was eluted into 50 µl of sterile water. Extractions were pooled by host species for sequencing. About 25 µl of each extraction was used in each pool, and this was concentrated using the NucleoSpin RNA Clean‑up XS, Micro kit for RNA clean up and concentration (Macherey-Nagel). The concentrated RNA was eluted into 20 µl of sterile water.

#### Plant material

RNA was extracted using the Rneasy plant mini extraction kit (Qiagen). The plant material in RNAlater was thawed just enough to remove approximately 20–30 mg. This was placed into a tube with a sterile stainless steel 6 mm bead. The tube, sample, bead and adapter set were then cooled at −80 °C for 30 min. After cooling, the plant tissue was disrupted by beating using the TissueLyser II (Qiagen) at 30 Hz for 2 min. The kit protocol was then followed for the remainder of the extraction. The RNA was eluted in 50 µl of sterile water. Extractions were pooled and concentrated as described above. Before concentrating, the pooled RNA was treated with DNase, using the rDNase Set (Macherey-Nagel).

#### Invertebrates

RNA was extracted using the RNeasy plus mini extraction kit (Qiagen). For small invertebrates (<30 mg), the whole body was used in the extraction. For larger invertebrates, a 30 mg piece of the abdomen was used. The frozen tissue was placed into a tube with a sterile stainless steel 6 mm bead, and 300–600 µl of buffer was added, depending on the amount of material. The tissue was disrupted by beating using the TissueLyser II (Qiagen) at 30 Hz for 4 min. The kit protocol was then followed for the remainder of the extraction. The RNA was eluted in 50 µl of sterile water. Extractions were pooled and concentrated as described above.

### Total RNA sequencing

Complementary DNA libraries were prepared using the Stranded Total RNA Prep with Ribo-Zero Plus (Illumina) for cloacal swabs and invertebrates and the TruSeq Stranded Total RNA with Ribo-Zero Plant (Illumina) for plants. Libraries were sequenced on the Illumina Novaseq platform at Auckland Genomics, University of Auckland, and the Australian Genome Research Facility, with invertebrates, plants and vertebrates sequenced entirely independently (that is, on different lanes and sequencing runs). One blank negative control library (that is, a sterile water and reagent mix) was sequenced with each sequencing run (one each for vertebrates, invertebrates and plants).

### Quality control, assembly and virus identification

Using Trimmomatic (0.38)^30^, adapters and bases below a quality of 5 were trimmed, using a sliding window approach with a window size of 4. Bases were cut if below a quality score of 3 at the beginning and end of the reads. Using bbduk in BBtools (bbmap 37.98)^31^, sequences less than 100 nucleotides in length or below an average quality of 10 were removed.

Reads were de novo assembled using Megahit (1.2.9)^32^. Viruses were identified by comparing the assembled contigs to the National Center for Biotechnology Information (NCBI) nucleotide database (nt) and non-redundant protein database (nr) using Blastn (blast+ 2.1.2)^33^ and Diamond Blastx (Diamond 2.0.9)^34^. Contigs were retained that had hits to viruses and an open reading frame greater than 300 nucleotides for nr hits (contig length range = 300–32,334). Sequence similarity cut-off values of 1 × 10^−^^5^ and 1 × 10^−^^10^ were used for the nt and nr databases, respectively, to prevent false positives. Virus transcript abundance was estimated using Bowtie2 (2.2.5)^35^. Viruses that met the following conditions were assumed to be contaminated as a result index-hopping from another library, and removed: (1) viruses were sequenced on the same lane, (2) the total read count was <0.1% of the read count in the other library, and (3) viruses were >99% identical at the nucleic acid level. Any virus found in the blank negative control libraries was assumed to have resulted from contamination and similarly removed from all libraries and analyses.

### Ecological analysis

All analyses were conducted in R (4.0.5)^36^. Viruses were grouped into viral families, as classified by the International Committee on Taxonomy of Viruses or NCBI. Viruses not classified to family level were included provided they had an order-level classification (for example, unclassified *Picornavirales* were included as a ‘family’). We did not group at a lower classification as it resulted in many viruses being excluded: only 56% of the viruses could be classified to the genus level, whereas 84% could be classified to the family level. An operational taxonomic unit table (Supplementary Data 1) was created using viral abundance expressed as the number of reads per million (RPM, that is, the number of reads from the virus family divided by the total number of reads in the library, multiplied by 1 million). Alpha diversity (richness and Shannon diversity) was calculated, and the role of host taxonomy and host diet were interrogated using previously described protocols^37^. Beta diversity (that is, shared diversity across host phyla) was visualized at the virus family level using non-metric multidimensional scaling with a Bray–Curtis dissimilarity matrix, presented as an ordination plot using the R packages phyloseq v1.34.0 (ref. ^38^) and scatterplot3d v0.3-41 (ref. ^39^). Bray–Curtis dissimilarity is a statistic ranging from 0 to 1 that reflects the dissimilarity of communities between libraries, with 0 meaning both libraries have an identical community, and 1 meaning the libraries have no virus families in common. Pairwise permutational analyses of variance (PERMANOVA, adonis2 in the vegan R package v2.5-7 (ref. ^40^) and pairwise PERMANOVA test from pairwiseAdonis v0.4 (ref. ^41^)) were used to test for differences in the community based on host phylogenetic groupings and host ecology, using the Bray–Curtis dissimilarity matrix and an alpha of 0.05 following a Bonferroni correction. Where multiple terms were used, the marginal effects of each term were tested using by=“margin” in adonis2.

To explore the effect of host phylogeny and diet on the viral community at lower taxonomic levels within Chordata, we conducted Mantel tests to determine the degree of correlation between three dissimilarity matrices—viral community (the Bray–Curtis dissimilarity matrix as described above), host relatedness and host ecological similarity—using the package vegan^40^. The host relatedness dissimilarity matrix was generated using the sum of branch lengths from a phylogenetic tree created using the Open Tree of Life and R package rotl v3.0.14 (ref. ^42^). The ecological similarity matrix was created using two host dietary factors, ‘eat invertebrates yes/no’ and ‘eat plants ‘yes/no’, combined into a matrix using the function vegdist in the package vegan^40^, with the Manhattan dissimilarity index. A partial Mantel test was used to test the correlatedness of ecological similarity and viral community while controlling for host relatedness.

A bipartite network was constructed using igraph^43^ and visualized using visNetwork^44^ based on the presence or absence of each virus family in each host library. Two sets of nodes were defined: virus families and host species. One link in the network corresponded to a virus node inhabiting a host node whenever the virus family was found in the host library. This host–virome network comprised 49 host libraries, 112 virus families and 926 interactions between these two node sets. Modules (that is, groups of nodes with more links among them than to the rest of the network, also named communities) were identified to infer the community structure in the host–virome network. To do this, we used the DIRTLPAwb+ community detection algorithm^45^ in the bipartite package^46^ that identifies partitions with high modularity scores by maximizing weighted modularity, weighted using log abundance (not RPM). DIRTLPAwb+ uses multiple iterations of the LPBwb+ algorithm, based on Barber’s modularity^45,47^ equation (1):

1 $$Q=1/2M\sum \big({A}_{{ij}}-{P}_{{ij}}\big){\delta }\big({\,g}_{i},{g}_{j}\big)$$

in which *Q* is the modularity score, *m* is the number of links in the network, and *g*_*i*_ and *g*_*j*_ are the assigned modules for nodes *i* and *j*. *Aij* = 1 if a link exists between nodes *i* and *j*, or 0 if no link. *P*_*ij*_ is the probability that a link exists between nodes *i* and *j* based on a null model. *δ*(*g*_*i*_,*g*_*j*_) = 1 if the modules are the same, and 0 if different^44,46^.

Pairwise PERMANOVA tests were used to test for differences in community composition between modules, using module as an independent factor and an alpha of 0.05 following Bonferroni correction.

We evaluated the robustness of the modules identified by rarefying to the lowest sequencing depth following the methods used by Lurgi et al.^48^. We performed pairwise PERMANOVA tests on 100 rarefied data sets of the original data using the modules detected in our host–virome network as an independent variable. We rarefied the data to the size of the smallest library (630 contigs) using the rrarefy function of the vegan package^40^. This was repeated 100 times independently to obtain 100 different rarefied data sets, using the replicate function in base-R v4.0.5. We then analysed each of these rarefied data sets with the pairwise PERMANOVA test and averaged the result across the 100 rarefied data sets. In this way, we tested whether the communities identified by the modularity analysis remained consistently significantly different when rarefied down to the lowest sequencing depth.

Using the modules obtained from the community detection algorithm, we evaluated the roles of individual species in the network by analysing the degree distribution (the distribution of the number of links from each node). To assess the distribution of links between nodes in the host–virome network, we also calculated the cumulative degree distribution and fitted a truncated power law, using nls and the Kolmogorov–Smirnov test to evaluate whether it fitted the power law distribution. The truncated power law distribution was as follows in equation (2):

2 $${Pc}\left(k\right)={k}^{\,i}\,{k}^{-(k/z)}$$

where *k* is the cumulative degree distribution, *i* is the power-law decay exponent and *k*^*−*(*k*/*z*)^ is the exponential cut-off for the truncation, where *z* is the cut-off value beyond which the power-law distribution no longer fits. We then compared this distribution to a null model of a random bipartite graph created using the Erdos–Renyi model, with the same number of host and virus nodes and interactions but with the interactions randomized. The impact of host taxonomy and host diet on node level properties of the network (degree, betweenness and eigenvector centrality) were interrogated using general linear models.

To examine the strength of connections between hosts created by shared viromes, we created a unipartite network (that is, links between hosts with shared virus families) based on the Bray–Curtis dissimilarity matrix, using the phyloseq v1.34.0 and igraph v1.2.11 R packages. Given the Bray–Curtis statistic ranges from 0 to 1, a cut-off was required to determine how similar two communities need to be to link them in the network. We chose a cut-off of 0.9, which is the lowest that still creates a cohesive network (that is, no isolated groups of nodes). However, this cut-off does create nine singletons (nodes with no connections), which were removed. The network (referred to as the ‘host community’ network) comprised 40 hosts and 99 interactions. The ‘small world’ properties of this network were examined using the distance_table functions in igraph, including calculating the shortest paths (the smallest number of links between each node) and average shortest path length (the mean of all the shortest paths). We used the smallworldness function in qgraph v1.9.2 (ref. ^49^), which uses the transitivity (the probability that the adjacent nodes of a vertex are connected, using the definition and formula developed by Barrat et al.^50^) and average shortest path length to compare the network to 1,000 randomly generated networks. This generates a ‘small-worldness’ index as developed by Humphries and Gurney^51^, which determines whether the network is significantly different from 1,000 randomly generated null networks and whether the network can be deemed a ‘small world’.

### Phylogenetic analysis

The patterns of virus diversity within viral families were visualized using phylogenetic trees with one viral family per module examined in detail, out of a total of 112 viral families. Amino acid sequences were aligned using MAFFT (7.402)^52^ with the L-INS-i algorithm and trimmed with a gap threshold of 0.9 and at least 20% of the sequence conserved using TrimAl (1.4.1)^53^. Individual maximum likelihood phylogenetic trees for each virus family were estimated using IQ-TREE (1.6.12)^54^, with the best-fit substitution model determined by the program and node robustness assessed by using the approximate likelihood ratio test with 1,000 replicates. Phylogenetic trees were visualized using APE (5.4)^55^ and ggtree (2.4.1)^56^ in R.

### Reporting summary

Further information on research design is available in the Nature Portfolio Reporting Summary linked to this article.

### Supplementary information

Supplementary Information Supplementary Tables 1–7.

Reporting Summary

Supplementary Data 1 Operational taxonomic unit table of raw virus abundance levels.

## Data Availability

The operational taxonomic unit table used in analyses is provided in Supplementary Data 1. The non-host sequence data generated in this study has been deposited in the Sequence Read Archive (SRA) under the accession number SAMN30927701-49. Virus consensus sequences have been submitted to NCBI/GenBank and assigned accession numbers OQ986602–OQ987814.

## References

[1] Geoghegan JL, Duchêne S, Holmes EC (2017). Comparative analysis estimates the relative frequencies of co-divergence and cross-species transmission within viral families. PLoS Pathog..

[2] Zhang Y-Z, Chen Y-M, Wang W, Qin X-C, Holmes EC (2019). Expanding the RNA virosphere by unbiased metagenomics. Annu. Rev. Virol..

[3] Holmes EC (2022). The ecology of viral emergence. Annu. Rev. Virol..

[4] French RK, Holmes EC (2020). An ecosystems perspective on virus evolution and emergence. Trends Microbiol..

[5] Streicker DG (2010). Host phylogeny constrains cross-species emergence and establishment of rabies virus in bats. Science.

[6] Olival KJ (2017). Host and viral traits predict zoonotic spillover from mammals. Nature.

[7] Harvey E, Holmes EC (2022). Diversity and evolution of the animal virome. Nat. Rev. Microbiol..

[8] Luis AD (2015). Network analysis of host–virus communities in bats and rodents reveals determinants of cross‐species transmission. Ecol. Lett..

[9] Holmes EC (2022). COVID-19—lessons for zoonotic disease. Science.

[10] Weitz JS (2015). A multitrophic model to quantify the effects of marine viruses on microbial food webs and ecosystem processes. ISME J..

[11] Zayed AA (2022). Cryptic and abundant marine viruses at the evolutionary origins of Earth’s RNA virome. Science.

[12] Haynes KJ (2009). Spatial synchrony propagates through a forest food web via consumer–resource interactions. Ecology.

[13] McLean IG (1987). Mixed-species flocking of forest birds on Little Barrier Island. N. Z. J. Zool..

[14] Watts DJ, Strogatz SH (1998). Collective dynamics of ‘small-world’ networks. Nature.

[15] Longdon B (2011). Host phylogeny determines viral persistence and replication in novel hosts. PLoS Pathog..

[16] Gupta P, Vishnudas C, Robin V, Dharmarajan G (2020). Host phylogeny matters: examining sources of variation in infection risk by blood parasites across a tropical montane bird community in India. Parasit. Vectors.

[17] Souza DT (2017). Analysis of bacterial composition in marine sponges reveals the influence of host phylogeny and environment. FEMS Microbiol. Ecol..

[18] Li L (2010). Bat guano virome: predominance of dietary viruses from insects and plants plus novel mammalian viruses. J. Virol..

[19] Forni D (2018). Ancient evolution of mammarenaviruses: adaptation via changes in the L protein and no evidence for host–virus codivergence. Genome Biol. Evol..

[20] Ramsden C, Holmes EC, Charleston MA (2009). Hantavirus evolution in relation to its rodent and insectivore hosts: no evidence for codivergence. Mol. Biol. Evol..

[21] Minot S (2011). The human gut virome: inter-individual variation and dynamic response to diet. Genome Res..

[22] Wille M (2018). Virus–virus interactions and host ecology are associated with RNA virome structure in wild birds. Mol. Ecol..

[23] Geoghegan JL (2021). Virome composition in marine fish revealed by meta-transcriptomics. Virus Evol..

[24] Montoya JM, Pimm SL, Solé RV (2006). Ecological networks and their fragility. Nature.

[25] Montoya JM, Solé RV (2002). Small world patterns in food webs. J. Theor. Biol..

[26] Wille M, Geoghegan JL, Holmes EC (2021). How accurately can we assess zoonotic risk?. PLoS Biol..

[27] Valverde S (2018). The architecture of mutualistic networks as an evolutionary spandrel. Nat. Ecol. Evol..

[28] Jordano P, Bascompte J, Olesen JM (2003). Invariant properties in coevolutionary networks of plant–animal interactions. Ecol. Lett..

[29] Williams RJ, Berlow EL, Dunne JA, Barabási A-L, Martinez ND (2002). Two degrees of separation in complex food webs. Proc. Natl Acad. Sci. USA.

[30] Bolger AM, Lohse M, Usadel B (2014). Trimmomatic: a flexible trimmer for Illumina sequence data. Bioinformatics.

[31] Bushnell, B. *BBMap Short Read Aligner* (Univ. California, 2016); https://www.sourceforge.net/projects/bbmap

[32] Li D, Liu C-M, Luo R, Sadakane K, Lam T-W (2015). MEGAHIT: an ultra-fast single-node solution for large and complex metagenomics assembly via succinct de Bruijn graph. Bioinformatics.

[33] Camacho C (2009). BLAST+: architecture and applications. BMC Bioinform..

[34] Buchfink B, Xie C, Huson DH (2015). Fast and sensitive protein alignment using DIAMOND. Nat. Methods.

[35] Langmead B, Salzberg S (2013). Bowtie2. Nat. Methods.

[36] R Core Team. *R: A Language and Environment for Statistical Computing* (R Foundation for Statistical Computing, 2021).

[37] Chen Y-M (2022). RNA viromes from terrestrial sites across China expand environmental viral diversity. Nat. Microbiol..

[38] McMurdie PJ, Holmes S (2013). phyloseq: an R package for reproducible interactive analysis and graphics of microbiome census data. PLoS ONE.

[39] Ligges, U. & Mächler, M. *Scatterplot3d—An R Package for Visualizing Multivariate Data* Universität Dortmund, Sonderforschungsbereich 475 - Komplexitätsreduktion in Multivariaten Datenstrukturen, Dortmund (2002).

[40] Oksanen, J. et al. vegan: Community Ecology Package. R package version 2.5-7 *J. Veg. Sci.* (2020).

[41] Martinez Arbizu, P. *pairwiseAdonis: Pairwise Multilevel Comparison Using adonis. R package version 0.3* (2017).

[42] Michonneau F, Brown JW, Winter DJ (2016). rotl: an R package to interact with the Open Tree of Life data. Methods Ecol. Evol..

[43] Csardi G, Nepusz T (2006). The igraph software package for complex network research.. Int. J Complex Syst..

[44] Almende, B. Thieurmel, B. & Robert, T. *visNetwork: Network Visualization Using’vis. js’ Library. R package version* 2 CRAN (2019).

[45] Beckett SJ (2016). Improved community detection in weighted bipartite networks. R. Soc. Open Sci..

[46] Dormann CF, Gruber B, Fründ J (2008). Introducing the bipartite package: analysing ecological networks. R. News.

[47] Barber MJ (2007). Modularity and community detection in bipartite networks. Phys. Rev. E.

[48] Lurgi M, Thomas T, Wemheuer B, Webster NS, Montoya JM (2019). Modularity and predicted functions of the global sponge-microbiome network. Nat. Commun..

[49] Epskamp S, Cramer AO, Waldorp LJ, Schmittmann VD, Borsboom D (2012). qgraph: Network visualizations of relationships in psychometric data. J. Stat. Softw..

[50] Barrat A, Barthelemy M, Pastor-Satorras R, Vespignani A (2004). The architecture of complex weighted networks. Proc. Natl Acad. Sci. USA.

[51] Humphries MD, Gurney K (2008). Network ‘small-world-ness’: a quantitative method for determining canonical network equivalence. PLoS ONE.

[52] Katoh K, Standley DM (2013). MAFFT multiple sequence alignment software version 7: improvements in performance and usability. Mol. Biol. Evol..

[53] Capella-Gutiérrez S, Silla-Martínez JM, Gabaldón T (2009). trimAl: a tool for automated alignment trimming in large-scale phylogenetic analyses. Bioinformatics.

[54] Nguyen L-T, Schmidt HA, Von Haeseler A, Minh BQ (2015). IQ-TREE: a fast and effective stochastic algorithm for estimating maximum-likelihood phylogenies. Mol. Biol. Evol..

[55] Paradis E, Schliep K (2019). ape 5.0: an environment for modern phylogenetics and evolutionary analyses in R. Bioinformatics.

[56] Yu G, Smith DK, Zhu H, Guan Y, Lam TTY (2017). ggtree: an R package for visualization and annotation of phylogenetic trees with their covariates and other associated data. Methods Ecol. Evol..

