# Supplementary material for: Host phylogeny shapes viral transmission networks in an island ecosystem
Source: Nat Ecol Evol. 2023 Sep 7;7(11):1834–43. doi: 10.1038/s41559-023-02192-9 (PMC10627826; doi:10.1038/s41559-023-02192-9)
Supplement: Supplementary file 1 — Supplementary Tables 1–7. [file 41559_2023_2192_MOESM1_ESM.pdf]

nature ecology & evolution

Article

https://doi.org/10.1038/s41559-023-02192-9

Host phylogeny shapes viral transmission networks in an island ecosystem

In the format provided by the authors and unedited

Supplementary Table 1. Detailed information on each sequencing library. Chordate diet was determined using expert knowledge and published studies.

Latin name	Library code	Common name	Phylum	Class	Order	Family	Genus	Diet (chordates only)	Samples per library	Total reads	Viral abundance (RPM)	Richness (number of viral families)
Amphipoda spp.	AMPH	Landhopper	Arthropoda	Malacostraca	Amphipoda	Talitridae			10	110541625	48	13
Anthornis melanura	ANME	Bellbird / korimako	Chordata	Aves	Passeriformes	Meliphagidae	*Anthornis*	Omnivorous	10	229185897	9719	32
Apteryx owenii	APOW	Little spotted kiwi	Chordata	Aves	Apterygiformes	Apterygidae	*Apteryx*	Insectivorous	3	197211236	35	5
Araneae spp.	ARAN	Spider	Arthropoda	Arachnida	Araneae				10	95561385	4373	29
Blattodea spp.	BLAT	Cockroach	Arthropoda	Insecta	Blattodea	Blattidae	*Celatoblatta*		5	133850527	20	15
Poodytes punctatus	BOPU	Fernbird / mātātā	Chordata	Aves	Passeriformes	Locustellidae	*Poodytes*	Insectivorous	4	189786324	14896	23
Coleoptera spp.	COLE	Beetle	Arthropoda	Insecta	Coleoptera				10	128611425	5673	25
Cyanoramphus auriceps	CYAU	Kakariki / yellow-crowned parakeet	Chordata	Aves	Psittaciformes	Psittaculidae	*Cyanoramphus*	Omnivorous	6	239791291	263	8
Dicranoloma billardierei	DICBIL	Moss	Streptophyta	Bryopsida	Dicranales	Dicranaceae	*Dicranoloma*		9	150459100	16689	45
Diplura spp.	DIPL	Diplura	Arthropoda	Entognatha	Diplura				1	134084076	21	7
Diptera spp.	DIPT	Fly	Arthropoda	Insecta	Diptera				10	86664024	1970	14
Dracophyllum longifolium	DRALON	Dracophyllum	Streptophyta	Magnoliopsida	Ericales	Ericaceae	*Dracophyllum*		10	167202609	3838	35

Eudyptes pachyrhynchus	EUPA	Tawaki / Fiordland crested penguin	Chordata	Aves	Sphenisciformes	Spheniscidae	*Eudyptes*	Piscivorous	5	226372033	5458	26
Falco novaeseelandiae	FANO	New Zealand Falcon / kārearea	Chordata	Aves	Falconiformes	Falconidae	*Falco*	Carnivorous	1	201662827	4	5
Gerygone igata	GEIG	Grey warbler / riroriro	Chordata	Aves	Passeriformes	Acanthizidae	*Gerygone*	Insectivorous	9	226644582	407717	41
Geophilomorpha spp.	GEOP	Centipede	Arthropoda	Chilopoda	Geophilomorpha				1	127720386	137	13
Haplotaxida spp.	HAPL	Pot worm	Annelida	Clitellata	Haplotaxida	Enchytraeidae			4	149393198	3751	15
Hemiptera spp.	HEMI	Bug	Arthropoda	Insecta	Hemiptera				2	107380296	847	11
Hymenoptera spp.	HYME	Ant	Arthropoda	Insecta	Hymenoptera				5	99989785	23038	9
Isopoda spp.	ISOP	Slater	Arthropoda	Malacostraca	Isopoda				10	100171201	1721	16
Lepidoptera spp.	LEPI	Moth	Arthropoda	Insecta	Lepidoptera				2	115833281	24	11
Lepidothamnus intermedius	LEPINT	Yellow silver pine	Streptophyta	Pinopsida	Araucariales	Podocarpaceae	*Lepidothamnus*		10	145809339	1302	30
Lithobiomorpha spp.	LITH	Centipede	Arthropoda	Chilopoda	Lithobiomorpha				2	119449390	354	20
Mesostigmata spp.	MESO	Mite (pred)	Arthropoda	Arachnida	Mesostigmata				3	81062495	2089	14
Mohoua novaeseelandiae	MONO	Brown creeper / pīpipi	Chordata	Aves	Passeriformes	Mohouidae	*Mohoua*	Insectivorous	4	209799281	51828	28
Mohoua ochrocephala	MOOC	Mohua	Chordata	Aves	Passeriformes	Mohouidae	*Mohoua*	Insectivorous	10	187836029	12425	27
Nestor meridionalis	NEME	Kākā	Chordata	Aves	Psittaciformes	Strigopidae	*Nestor*	Omnivorous	1	215076937	64	7
Ninox novaeseelandiae	NINO	Morepork / ruru	Chordata	Aves	Strigiformes	Strigidae	*Ninox*	Insectivorous	1	646268	665	3
Nothofagus solandri	NOTSOL	Mountain beech	Streptophyta	Magnoliopsida	Fagales	Nothofagaceae	*Fuscospora*		10	124154845	2526	25
Oligosoma tekakahu	OLTE	Te Kakahu/Chalky Island skink	Chordata	Lepidosauria	Squamata	Scincidae	*Oligosoma*	Omnivorous	10	198227057	5635	20
Opiliones spp.	OPIL	Harvestman	Arthropoda	Arachnida	Opiliones				6	102508349	1386	21
Opisthopora spp.	OPIS	Earth worm	Annelida	Clitellata	Opisthopora				10	119919246	338	10

Oribatida spp.	ORIB	Mite	Arthropoda	Arachnida	Oribatida				6	75305102	3091	25
Orthoptera spp.	ORTH	Weta	Arthropoda	Insecta	Orthoptera	Anostostomatidae	*Hemiandrus*		1	134022234	595	5
Petroica australis	PEAU	South Island Robin / kakaruai	Chordata	Aves	Passeriformes	Petroicidae	*Petroica*	Insectivorous	10	221633876	60066	29
Petroica macrocephala	PEMA	Tomtit / miromiro	Chordata	Aves	Passeriformes	Petroicidae	*Petroica*	Insectivorous	5	213573349	129301	34
Philesturnus carunculatus	PHCA	Tieke / South Island Saddleback	Chordata	Aves	Passeriformes	Callaeidae	*Philesturnus*	Insectivorous	10	204580700	274434	30
Polydesmida spp.	POLYD	Millipede	Arthropoda	Diplopoda	Polydesmida				1	146389854	77	12
Pseudopanax crassifolius	PSECRA	Lancewood	Streptophyta	Magnoliopsida	Apiales	Araliaceae	*Pseudopanax*		9	159414298	440	28
Pseudoscorpiones spp.	PSEU	Pseudoscorpion	Arthropoda	Arachnida	Pseudoscorpiones				6	111900992	8980	36
Pterodroma inexpectata	PTIN	Kōrure / Mottled Petrel	Chordata	Aves	Procellariiformes	Procellariidae	*Pterodroma*	Piscivorous	10	242626127	14	3
Ardenna grisea	PUGR	Tītī / Sooty Shearwater	Chordata	Aves	Procellariiformes	Procellariidae	*Ardenna*	Piscivorous	10	195513371	195	25
Rhipidura fuliginosa	RHFU	Fantail / pīwakawaka	Chordata	Aves	Passeriformes	Rhipiduridae	*Rhipidura*	Insectivorous	10	249844003	426626	31
Ripogonum scandens	RIPSCA	Supplejack	Streptophyta	Magnoliopsida	Liliales	Ripogonaceae	*Ripogonum*		4	139857060	500	20
Scolopendromorpha spp.	SCOL	Centipede	Arthropoda	Chilopoda	Scolopendromorpha				1	86869494	41	4
Spirostreptida spp.	SPIR	Millipede	Arthropoda	Diplopoda	Spirostreptida				1	117989930	4911	17
Strigops habroptila	STHA	Kākāpō	Chordata	Aves	Psittaciformes	Strigopidae	*Strigops*	Herbivorous	10	241609810	651	8
Tricladida spp.	TRIC	Flatworm	Platyhelminthes	Rhabditophora	Tricladida	Geoplanidae	*Australopacifica*		1	74626559	112	6
Vespula germanica	WASP	German wasp	Arthropoda	Insecta	Hymenoptera	Vespidae	*Vespula*		4	91075333	10299	10

Supplementary Table 2. Results of permutational analysis of variance (PERMANOVA) models. These models were performed on a Bray-Curtis dissimilarity matrix, created from the OTU abundance table (the abundance of each virus family in each library). Abundance was the number of reads divided by the total reads per library, multiplied by one million (reads per million). Where there were multiple comparisons per model, a pairwise PERMANOVA was used. Significant comparisons (Bonferroni adjusted p-value <0.05) are shown with grey shading.

Independent variable(s)	Comparison	Degrees of freedom	Sums of squares	F model	R^2	p-value	adjusted p-value
Host phyla	Main effect	4	2.99	1.68	0.13	0.0001	0.0001
Host phyla	Chordata vs Arthropoda	1	0.82	1.79	0.04	0.003	0.03
	Chordata vs Streptophyta	1	1.16	2.73	0.11	0.001	0.01
	Chordata vs Annelida	1	0.66	1.50	0.07	0.03	0.3
	Chordata vs Platyhelminthes	1	0.54	1.23	0.06	0.05	0.5
	Arthropoda vs Streptophyta	1	0.92	2.03	0.08	0.001	0.01
	Arthropoda vs Annelida	1	0.56	1.19	0.05	0.05	0.5
	Arthropoda vs Platyhelminthes	1	0.48	1.00	0.05	0.5	1
	Streptophyta vs Annelida	1	0.77	2.15	0.26	0.04	0.4
	Streptophyta vs Platyhelminthes	1	0.59	1.63	0.25	0.1	1
	Annelida vs Platyhelminthes	1	0.55	1.53	0.61	0.3	1
Host class	Main effect	12	6.75	1.28	0.30	0.0001	0.0001
Host order	Main effect	36	18.21	1.37	0.80	0.0001	0.0001

Host order + host diet (chordates only)	Insectivore vs non-insectivore	1	0.49	1.54	0.06	0.01	0.03
	Plant-eater vs non-plant-eater	1	0.56	1.75	0.07	0.0008	0.002
Module	4 vs 3	1	0.78	2.87	0.12	0.002	0.012
	4 vs 1	1	1.66	6.21	0.16	0.001	0.006
	4 vs 2	1	1.10	4.74	0.18	0.001	0.006
	3 vs 1	1	0.98	3.56	0.13	0.001	0.006
	3 vs 2	1	0.90	4.13	0.24	0.001	0.006
	1 vs 2	1	1.37	5.79	0.19	0.001	0.006

Supplementary Table 3. The mean (± standard deviation) of permutational analysis of variance (PERMANOVA) models over 100 rarefied data sets of the original OTU abundance table, with module number as the independent variable. Abundance was the natural log of the raw abundances.

Comparison	Degrees of freedom	Mean Sums of squares (±SD)	Mean F model (±SD)	Mean R2 (±SD)	mean p-value (±SD)	mean adjusted p-value (±SD)
4 vs 3	1	0.80 (0.04)	2.65 (0.15)	0.11 (0.005)	0.006 (0.003)	0.04 (0.02)
4 vs 1	1	1.61 (0.06)	4.90 (0.19)	0.13 (0.004)	0.001 (0)	0.006 (0)
4 vs 2	1	1.84 (0.06)	6.83 (0.28)	0.24 (0.007)	0.001 (0.0001)	0.006 (0.0006)
3 vs 1	1	0.90 (0.06)	2.47 (0.13)	0.01 (0.005)	0.002 (0.001)	0.01 (0.007)
3 vs 2	1	1.02 (0.04)	3.47 (0.21)	0.21 (0.01)	0.002 (0.0008)	0.01 (0.005)
1 vs 2	1	1.41 (0.04)	4.20 (0.18)	0.15 (0.005)	0.001 (0.0001)	0.006 (0.0008)

.

Supplementary Table 4. The results of general linear models comparing the effects of host taxonomy and host diet on network node level properties within the host phyla Chordata. Eigen = eigenvector centrality.

Model	Comparison	Degrees of freedom	Deviance	p-value
Degree ~ Host order + host diet (insectivore)	Host order	7	2304	6.32e-09
	Insectivore vs. non-insectivore	1	0.17	0.95
Degree ~ Host order + host diet (herbivore)	Host order	7	2304	5.628e-09
	Plant-eater vs. non-plant eater	1	2.35	0.82
Betweenness ~ Host order + host diet (insectivore)	Host order	7	722458	0.11
	Insectivore vs. non-insectivore	1	138	0.96
Betweenness ~ Host order + host diet (herbivore)	Host order	7	722458	0.029
	Plant-eater vs. non-plant eater	1	151725	0.07

Eigen ~ Host order + host diet (insectivore)	Host order	7	1.59	<2e-16
	Insectivore vs. non-insectivore	1	0.0005	0.86
Eigen ~ Host order + host diet (herbivore)	Host order	7	1.59	<2e-16
	Plant-eater vs. non-plant eater	1	0.001	0.78

Supplementary Table 5. The hosts and viruses belonging to each module, as identified by the modularity analysis. Shading represents the four different modules and corresponds to the colours used in Figure 3.

Module	Hosts (library code)	Virus families
Module 1	COLE	*Adenoviridae*
	HEMI	*Adintoviridae*
	GEOP	*Aliusviridae*
	LITH	*Artoviridae*
	BLAT	*Baculoviridae*
	DIPL	*Benyviridae*
	OPIS	*Chuviridae*
	TRIC	*Circoviridae*
	DIPT	*Euroniviridae*
	OPIL	*Iridoviridae*
	AMPH	*Lispiviridae*
	POLYD	*Metaviridae*
	SPIR	*Mimiviridae*
	ORIB	*Mononiviridae*
	LEPI	*Nairoviridae*
	HAPL	*Nudiviridae*
	ARAN	*Nyamiviridae*

	ORTH	*Orthomyxoviridae*
		Parvoviridae
		Peribunyaviridae
		Phasmaviridae
		Phenuiviridae
		Polyomaviridae
		Poxviridae
		Rhabdoviridae
		Tospoviridae
		Totiviridae
		Unclassified Martellivirales
		Unclassified Mononegavirales
		Xinmoviridae
Module 2	DRALON	*Amalgaviridae*
	PSECRA	*Arenaviridae*
	MESO	*Aspiviridae*
	DICBIL	*Barnaviridae*
	NOTSOL	*Betaflexiviridae*
	PSEU	*Birnaviridae*
	RIPSCA	*Botourmiaviridae*
	LEPINT	*Bromoviridae*
		Caulimoviridae

Chrysoviridae
Closteroviridae
Deltaflexiviridae
Endornaviridae
Fusariviridae
Geminiviridae
Genomoviridae
Herpesviridae
Hypoviridae
Kitaviridae
Megabirnaviridae
Mitoviridae
Mymonaviridae
Narnaviridae
Partitiviridae
Phycodnaviridae
Pithoviridae
Polymycoviridae
Potyviridae
Qinviridae
Rountreeviridae
Tectiviridae

		Togaviridae
		Unclassified Bunyavirales
		Unclassified Tymovirales
		Virgaviridae
		Yueviridae
Module 3	FANO	*Atkinsviridae*
	NEME	*Fiersviridae*
	STHA	*Hepadnaviridae*
	OLTE	*Herelleviridae*
	ISOP	*Inoviridae*
	PUGR	*Leviviridae*
	EUPA	*Marnaviridae*
		Microviridae
		Myoviridae
		Nimaviridae
		Picobirnaviridae
		Podoviridae
		Sarthroviridae
		Siphoviridae
		Steitzviridae
		Unclassified Caudovirales
		Unclassified Ortervirales

Module 4	HYME	*Alphatetraviridae*
	ANME	*Anelloviridae*
	MONO	*Astroviridae*
	SCOL	*Autographiviridae*
	RHFU	*Caliciviridae*
	BOPU	*Carmotetraviridae*
	WASP	*Dicistroviridae*
	GEIG	*Flaviviridae*
	CYAU	*Hepeviridae*
	APOW	*Iflaviridae*
	MOOC	*Luteoviridae*
	NINO	*Mayoviridae*
	PTIN	*Mesoniviridae*
	PEAU	*Nodaviridae*
	PHCA	*Permutotetraviridae*
	PEMA	*Picornaviridae*
		Polycipiviridae
		Polydnaviridae
		Reoviridae
		Retroviridae
		Schitoviridae
		Secoviridae

Sinhaliviridae
Solemoviridae
Solinviviridae
Tombusviridae
Tymoviridae
Unclassified Nidovirales
Unclassified Picornavirales

Supplementary Table 6. Details of the sequence alignments used to estimate the phylogenetic trees for each of the four viral families chosen for phylogenetic analysis out of a total of 112 viral families. The % pairwise identity refers to the percentage of pairwise residues that are identical in the alignment, excluding gap-gap residues.

**Family**	**Protein**	**Alignment length (amino acids)**	**Number of sequences**	**% Pairwise Identity**
Parvoviridae	Non-structural protein 1	584	292	15.8
Caulimoviridae	Polyprotein	938	123	40.9
Fiersviridae	RNA-dependent RNA polymerase	453	336	32.2
Caliciviridae	Polyprotein	1111	68	20.1

Supplementary Table 7. The abundance (expressed as reads per million) and percentage abundance of viruses from each host phylum for one viral family per module.

**Host phylum**	***Parvoviridae* (module 1)**	***Caulimoviridae* (module 2)**	***Fiersviridae* (module 3)**	***Caliciviridae* (module 4)**
Arthropoda	351 (99%)	0 (0%)	0 (0%)	1 (0.03%)
Chordata	2 (0.6%)	2 (0.2%)	10 (99%)	3952 (99.98%)
Streptophyta	0.1 (0.03%)	918 (99.8%)	0.1 (1%)	0 (0%)
Annelida	3 (0.3%)	0 (0%)	0 (0%)	0 (0%)
Platyhelminthes	0 (0%)	0	0 (0%)	0 (0%)
