# Supplementary material for: Host phylogeny shapes viral transmission networks in an island ecosystem
Source: Nat Ecol Evol. 2023 Sep 7;7(11):1834–43. doi: 10.1038/s41559-023-02192-9 (PMC10627826; doi:10.1038/s41559-023-02192-9)
Supplement: Supplementary file 2 — Reporting Summary [file 41559_2023_2192_MOESM2_ESM.pdf]

Corresponding author(s): Prof. Edward C. Holmes

Last updated by author(s): Jul 3, 2023

Reporting Summary

Nature Portfolio wishes to improve the reproducibility of the work that we publish. This form provides structure for consistency and transparency in reporting. For further information on Nature Portfolio policies, see our Editorial Policies and the Editorial Policy Checklist.

Statistics

For all statistical analyses, confirm that the following items are present in the figure legend, table legend, main text, or Methods section.

n/a	Confirmed	
☐	☒	The exact sample size (*n*) for each experimental group/condition, given as a discrete number and unit of measurement
☒	☐	A statement on whether measurements were taken from distinct samples or whether the same sample was measured repeatedly
☐	☒	The statistical test(s) used AND whether they are one- or two-sided<br>*Only common tests should be described solely by name; describe more complex techniques in the Methods section.*
☐	☒	A description of all covariates tested
☐	☒	A description of any assumptions or corrections, such as tests of normality and adjustment for multiple comparisons
☐	☒	A full description of the statistical parameters including central tendency (e.g. means) or other basic estimates (e.g. regression coefficient) AND variation (e.g. standard deviation) or associated estimates of uncertainty (e.g. confidence intervals)
☐	☒	For null hypothesis testing, the test statistic (e.g. *F*, *t*, *r*) with confidence intervals, effect sizes, degrees of freedom and *P* value noted<br>*Give P values as exact values whenever suitable.*
☒	☐	For Bayesian analysis, information on the choice of priors and Markov chain Monte Carlo settings
☐	☒	For hierarchical and complex designs, identification of the appropriate level for tests and full reporting of outcomes
☐	☒	Estimates of effect sizes (e.g. Cohen's *d*, Pearson's *r*), indicating how they were calculated

Our web collection on statistics for biologists contains articles on many of the points above.

Software and code

Policy information about availability of computer code

Data collection	No software was used for data collection
Data analysis	Software (all open source):<br>Trimmomatic (0.38)<br>BBtools (bbmap 37.98)<br>Megahit (1.2.9)<br>blast+ (2.1.2)<br>Diamond (2.0.9)<br>Bowtie2 (2.2.5)<br>R (4.0.5)<br>MAFFT (7.402)<br>TrimAl (1.4.1)<br>IQ-TREE (1.6.12)<br><br>R packages (all open source):<br>phyloseq (1.34.0)<br>scatterplot3d (0.3-41)<br>pairwiseAdonis (0.4)<br>rotl (3.0.12)<br>igraph (1.2.11)<br>Visnetwork (2.1.0)

bipartite (2.16)
vegan (2.5-7)
qgraph (1.9.2)
APE (5.4)
ggtree (2.4.1)

For manuscripts utilizing custom algorithms or software that are central to the research but not yet described in published literature, software must be made available to editors and reviewers. We strongly encourage code deposition in a community repository (e.g. GitHub). See the Nature Portfolio guidelines for submitting code & software for further information.

Data

Policy information about availability of data

All manuscripts must include a data availability statement. This statement should provide the following information, where applicable:

- Accession codes, unique identifiers, or web links for publicly available datasets
- A description of any restrictions on data availability
- For clinical datasets or third party data, please ensure that the statement adheres to our policy

The operational taxonomic unit table used in analyses is provided in Supplementary Data 1. The non-host sequence data generated in this study has been deposited in the Sequence Read Archive (SRA) under the accession numbers SAMN30927701-49. Virus consensus sequences have been submitted to NCBI/GenBank and assigned accession numbers OQ986602 - OQ987814.

Research involving human participants, their data, or biological material

Policy information about studies with human participants or human data. See also policy information about sex, gender (identity/presentation), and sexual orientation and race, ethnicity and racism.

Reporting on sex and gender	*Use the terms sex (biological attribute) and gender (shaped by social and cultural circumstances) carefully in order to avoid confusing both terms. Indicate if findings apply to only one sex or gender; describe whether sex and gender were considered in study design; whether sex and/or gender was determined based on self-reporting or assigned and methods used.*<br>*Provide in the source data disaggregated sex and gender data, where this information has been collected, and if consent has been obtained for sharing of individual-level data; provide overall numbers in this Reporting Summary. Please state if this information has not been collected.*<br>*Report sex- and gender-based analyses where performed, justify reasons for lack of sex- and gender-based analysis.*
Reporting on race, ethnicity, or other socially relevant groupings	*Please specify the socially constructed or socially relevant categorization variable(s) used in your manuscript and explain why they were used. Please note that such variables should not be used as proxies for other socially constructed/relevant variables (for example, race or ethnicity should not be used as a proxy for socioeconomic status).*<br>*Provide clear definitions of the relevant terms used, how they were provided (by the participants/respondents, the researchers, or third parties), and the method(s) used to classify people into the different categories (e.g. self-report, census or administrative data, social media data, etc.)*<br>*Please provide details about how you controlled for confounding variables in your analyses.*
Population characteristics	*Describe the covariate-relevant population characteristics of the human research participants (e.g. age, genotypic information, past and current diagnosis and treatment categories). If you filled out the behavioural & social sciences study design questions and have nothing to add here, write "See above."*
Recruitment	*Describe how participants were recruited. Outline any potential self-selection bias or other biases that may be present and how these are likely to impact results.*
Ethics oversight	*Identify the organization(s) that approved the study protocol.*

Note that full information on the approval of the study protocol must also be provided in the manuscript.

Field-specific reporting

Please select the one below that is the best fit for your research. If you are not sure, read the appropriate sections before making your selection.

☐ Life sciences ☐ Behavioural & social sciences ☒ Ecological, evolutionary & environmental sciences

For a reference copy of the document with all sections, see nature.com/documents/nr-reporting-summary-flat.pdf

Ecological, evolutionary & environmental sciences study design

All studies must disclose on these points even when the disclosure is negative.

Study description	We used metatranscriptomic (i.e., total RNA) sequencing to document the virome of an island community in New Zealand. Our sampling of the Pukenui/Anchor Island forest community included all key vertebrate species in addition to representative sampling of invertebrates and plants. Individual samples were pooled by species (for vertebrates and plants) and by phylogenetic order for

	invertebrates. The number of individual animals in each pool ranged from 1-10.
Research sample	A description of each of the species sampled is provided in Supplementary Table 1.
Sampling strategy	The sample size was determined by how many animals were caught in a 4 week period, to a maximum of 10, in line with our permit conditions. As this is a viral discovery project the prevalence of any virus in the population is inherently unknown such that it is impossible to do a sample size power calculation.
Data collection	Data was collected by Dr. Rebecca French (first author). Data collection included cloacal swabs (for vertebrates), plant tissue and invertebrate body tissue.
Timing and spatial scale	Sample collection was undertaken between the 17th of February and 14th of March 2021, with no gap in collection period.
Data exclusions	Viruses that met the following conditions: (i) sequenced on the same lane, (ii) the total read count was < 0.1% of the read count in the other library, and (iii) were >99% identical at the nucleic acid level were assumed to be contamination due to index-hopping from another library and removed. Any virus found in the blank negative control libraries was assumed to have resulted from contamination and similarly removed from all libraries and analyses. This exclusion criteria was pre-established.
Reproducibility	This study was a single snapshot of an island virome over a 4 week sampling period.
Randomization	Samples were grouped according to their taxonomy, therefore randomization is not relevant.
Blinding	We cannot determine what viruses will be detected during sample collection.

Did the study involve field work? ☒ Yes ☐ No

Field work, collection and transport

Field conditions	Temperate rainforest during late summer - early autumn (southern hemisphere).
Location	Pukenui Anchor Island, New Zealand 45°45'30.0"S 166°31'00.0"E
Access & import/export	This research was conducted under a Department of Conservation Wildlife Act Authority Authorisation number 86173-FAU, Authority for research and/or collection of material on public conservation land Authorisation number 86172-RES and had ethics approval from the University of Auckland reference number 002198.
Disturbance	Handling time of vertebrates was minimized. The researchers involved in capture and handling had a high level of experience.

Reporting for specific materials, systems and methods

We require information from authors about some types of materials, experimental systems and methods used in many studies. Here, indicate whether each material, system or method listed is relevant to your study. If you are not sure if a list item applies to your research, read the appropriate section before selecting a response.

Materials & experimental systems

n/a	Involved in the study	
☒	☐	Antibodies
☒	☐	Eukaryotic cell lines
☒	☐	Palaeontology and archaeology
☐	☒	Animals and other organisms
☒	☐	Clinical data
☒	☐	Dual use research of concern
☒	☐	Plants

Methods

n/a	Involved in the study	
☒	☐	ChIP-seq
☒	☐	Flow cytometry
☒	☐	MRI-based neuroimaging

Animals and other research organisms

Policy information about studies involving animals; ARRIVE guidelines recommended for reporting animal research, and Sex and Gender in Research

Laboratory animals	This study did not involve laboratory animals.
Wild animals	A list of species is provided in Supplementary Table 1. The 18 bird and 1 skink species were caught using four different methods, depending on the species in question. Small, flighted birds were caught using low canopy mist-netting, while larger flighted birds

	were caught with high canopy mist-nets. Bird calls were used to attract the birds to the area and into the nets. Non-flying birds were caught by hand or hand-net. Skinks were caught using gee-minnow traps. Once caught, the animals were weighed and a cloacal swab was taken. The animals were then released without being transported anywhere and without being held captive for any length of time.
Reporting on sex	The sex of the animals being sampled was not identified.
Field-collected samples	This study did not involve samples collected from the field.
Ethics oversight	This study had ethics approval from the University of Auckland reference number 002198.

Note that full information on the approval of the study protocol must also be provided in the manuscript.
